# Supplementary material for: Natural variation in BnaA9.NF-YA7 contributes to drought tolerance in Brassica napus L
Source: Nat Commun. 2024 Mar 7;15:2082. doi: 10.1038/s41467-024-46271-2 (PMC10920887; doi:10.1038/s41467-024-46271-2)
Supplement: Supplementary file 1 — Supplementary Information [file 41467_2024_46271_MOESM1_ESM.pdf]

Natural variation in *BnaA9.NF-YA7* contributes to drought tolerance in *Brassica napus* L.

Wang *et al.*

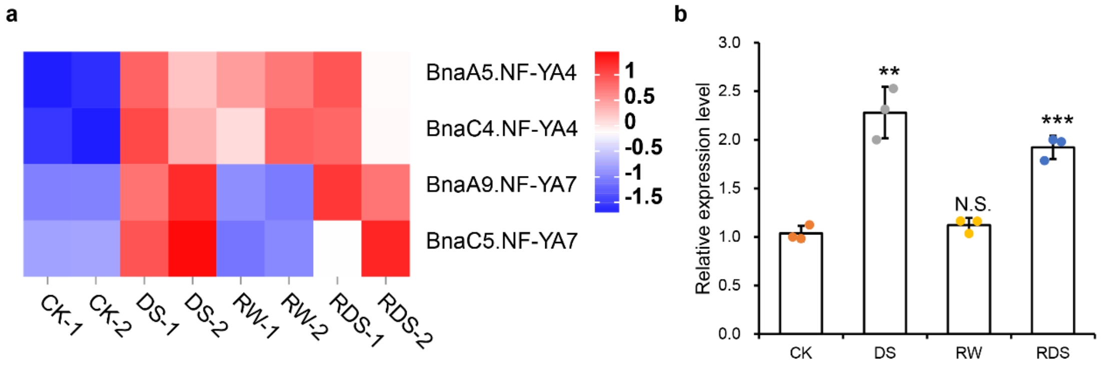

Supplementary Fig. 1. The expression level of *BnaA9.NF-YA7* was upregulated by drought stress. a, Heatmap of *BnaNF-YA7s* and *BnaNF-YA4s* under repeated drought-rewatering cycles. DS: drought stress; RW: rewatering; RDS: repeated drought stress. b, Real-time quantitative PCR (RT–qPCR) of B*naA9.NF-YA7* under repeated drying-wetting cycles. The values are means ± SD (n = 3 biological replicates). Asterisks indicate statistically significant differences based on two-tailed Student's t-test (**P < 0.01, ***P < 0.001, and $P = 1.47 \times 10^{-3}$ and 4.14×10^{-4} at DS and RDS). N.S., not significant.

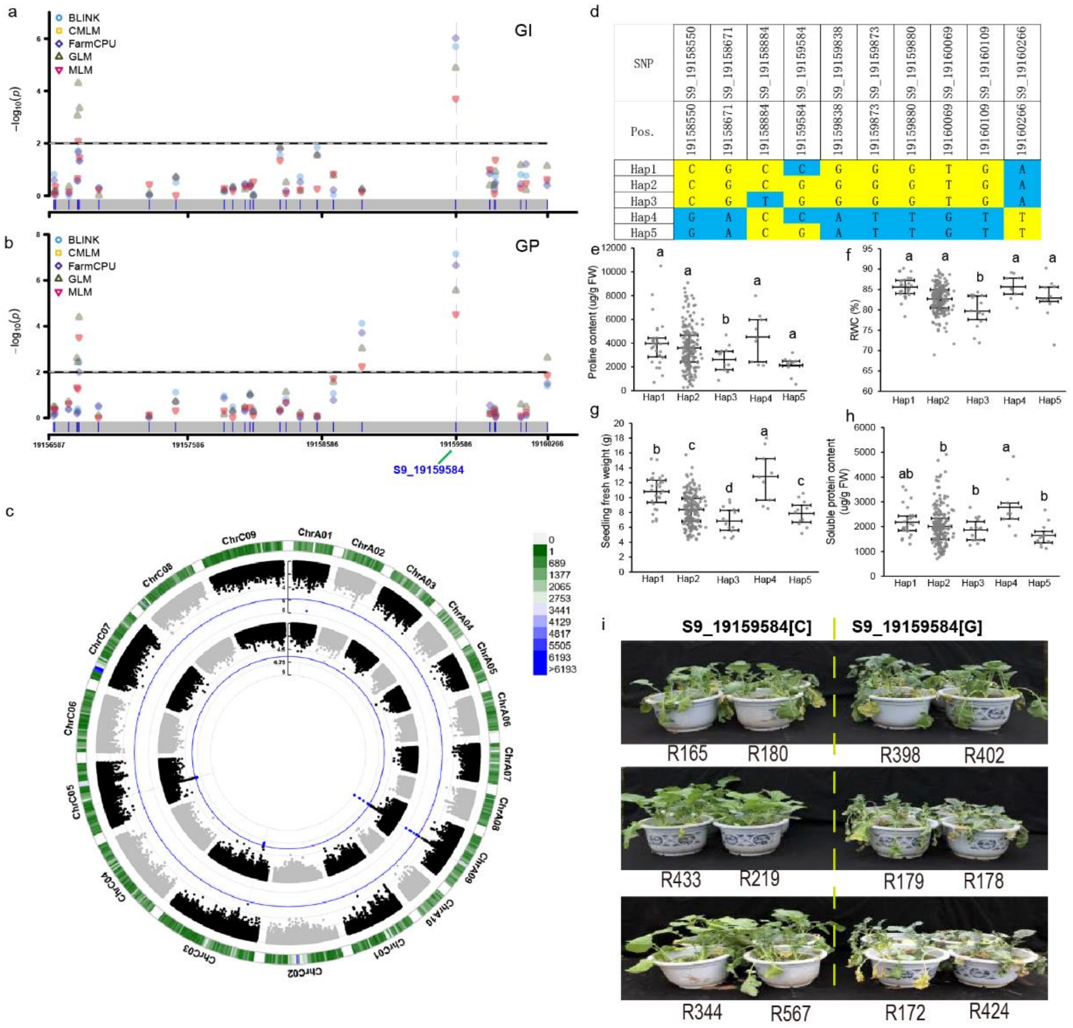

Supplementary Fig. 2. S9_19159584 is associated with drought tolerance. (**a** and **b**) Manhattan plot for the germination index (**a**) and germination percentage (**b**) of the *BnaA9.NF-YA7* gene locus. The dashed horizontal line depicts the significance threshold ($P = 1.0 \times 10^{-2}$), and the red dashed line indicates the significance of the SNP S9_19159584. **c,** Circular Manhattan plots of water holding capacity (WHC). From the inner ring to the outer ring are the results for 2016 and 2017. The red dots represent significant SNPs. **d,** Haplotypes in 608 accessions (haplotypes with fewer than three accessions were omitted) according to the SNP data. The region contained the coding region and was 2 kb upstream of the gene. (**e-h**) Comparison of proline content (**e**), relative water content (**f**), seedling fresh weight (**g**), and soluble protein content (**h**) among accessions of the five haplotypes (Hap1: n = 27 accessions; Hap2: n = 188 accessions; Hap3: n = 14 accessions; Hap4: n = 9 accessions; Hap5: n = 11 accessions;). The lowercase letters indicate significant differences ($P < 0.05$, One-way ANOVA followed by two-tailed LSD test). **i,** Phenotype of different varieties from two alleles. Source data are provided as a Source Data file.

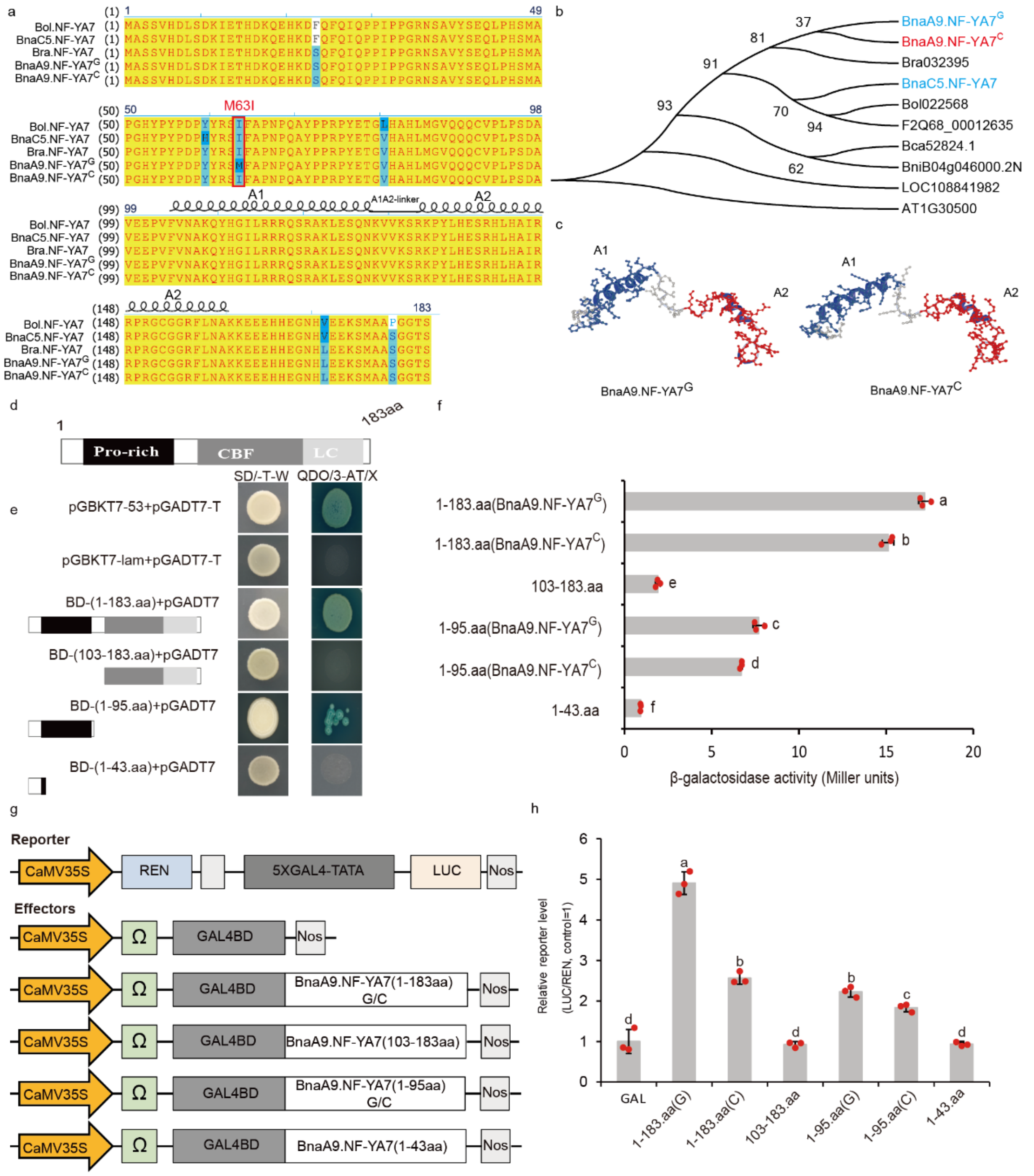

Supplementary Fig. 3. Characteristics of BnaA9.NF-YA7 protein. a, Multiple sequence alignment of NF-YA7 proteins from *B. napus*, *B. rapa*, and *B. oleracea*. The position shown in the red rectangular box corresponds to the M63I substitution. The A1 helix and A2 helix denote the NF-YB/C interaction domain and DNA-binding domain, respectively. **b,** Phylogenetic tree of NF-YA7 proteins in *A. thaliana*, *B. napus*, *B. rapa*, *B. oleracea*, *B. cretica*, *B. carinata*, *B. nigra*, and *R. sativus*. BnaA9.NF-YA7^{C} is highlighted in red. **c,** The tertiary structure of the BnaA9.NF-YA7^{G} and BnaA9.NF-YA7^{C} proteins. **d,** Schematic diagram of *BnaA9.NF-YA7*. Pro-rich, proline enrichment domain; CBF, CCAAT-binding factor; LC, pow complexity domain. Numbers indicate the positions of amino acid residues starting from the N-terminus. **e,** Yeast transcriptional activation assay testing for various truncated forms of *BnaA9.NF-YA7*. Transformed yeast cells were grown on nonselective SD/-L-W medium and selective QDO/3-AT/X medium. aa, amino acids. f, The β-galactosidase activity of yeast cells

grown on SD/-L-W medium. The values are means ± SD (n = 3 biological replicates). The lowercase letters indicate significant differences ($P < 0.05$, One-way ANOVA followed by two-tailed LSD test). **g**, Schematic diagram showing the constructs used in the transient transcriptional activity assays. **h**, The transcriptional activity of various truncated forms of BnaA9.NF-YA7 between Hap3 and Hap4. The values are means ± SD (n = 3 biological replicates). The lowercase letters indicate significant differences ($P < 0.05$, One-way ANOVA followed by two-tailed LSD test). Source data are provided as a Source Data file.

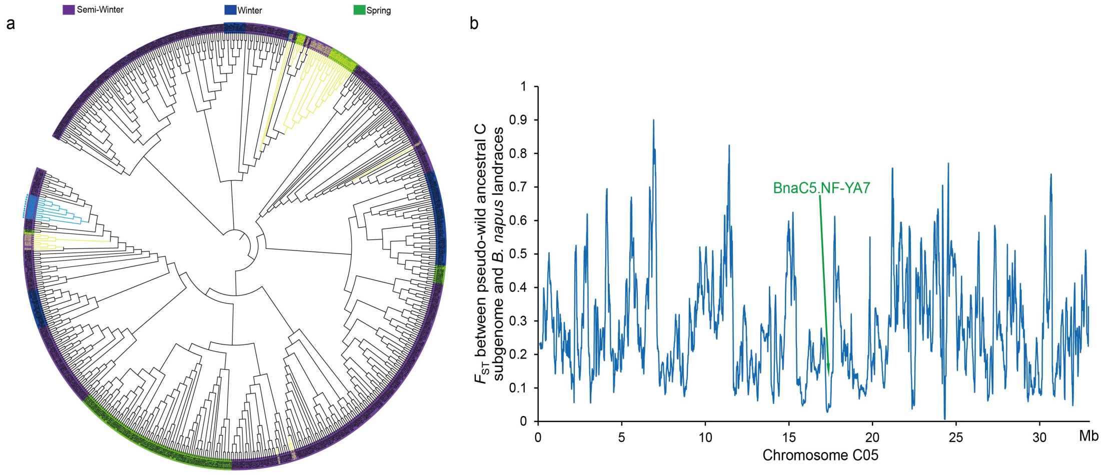

Supplementary Fig. 4. Phylogram of *BnaA9.NF-YA7*. a, Phylogram of *BnaA9.NF-YA7* generated from 608 diverse *B. napus* accessions (three main ecotypes: spring, winter, and semi-winter) showing the divergence between *BnaA9.NF-YA7*^{G} and *BnaA9.NF-YA7*^{C}. Yellow and red IDs represent Hap1 and Hap4, respectively. **b,** The level of population differentiation (F_{ST}) across chromosome C05 between *B. napus* and *B. oleracea*. Source data are provided as a Source Data file.

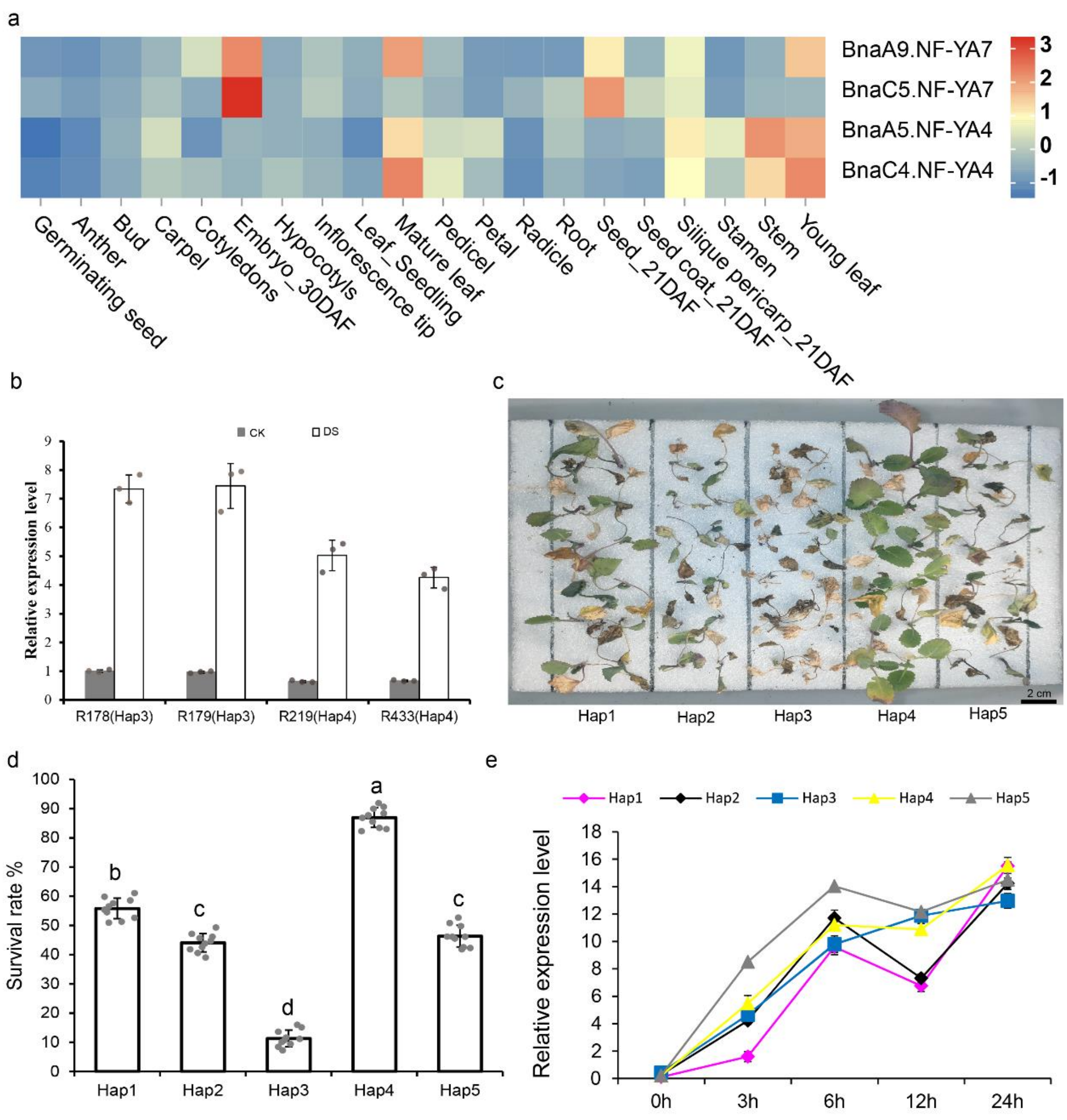

Supplementary Fig. 5. Phenotypes and expression levels of five haplotypes of *BnaA9.NF-YA7*. a, The relative expression of *BnaNF-YA7s* and *BnaNF-YA4s* from publicly available *B. napus* gene expression data (https://biodb.swu.edu.cn/brassica/) is presented as a heatmap, in which the RPKM (reads per kilobase of exon model per million mapped reads) values were transformed to log2 (value + 1). **b,** Expression levels of *BnaA9.NF-YA7* in Hap3 and Hap4 before and after drought treatment. The values are means ± SD (*n* = 3 biological replicates). (**c** and **d**) Phenotypes (**c**) and survival rates (**d**) of different varieties from five haplotypes under 15% PEG treatment. The values are means ± SD (*n* = 10 biologically independent experiments). The lowercase letters indicate significant differences ($P < 0.05$, One-way ANOVA followed by two-tailed LSD test). Scale bars = 2 cm. **e,** The expression level of *BnaC5.NF-YA7* in five haplotypes after drought treatment. The values are means ± SD (*n* = 3 biological replicates). Source data are provided as a Source Data file.

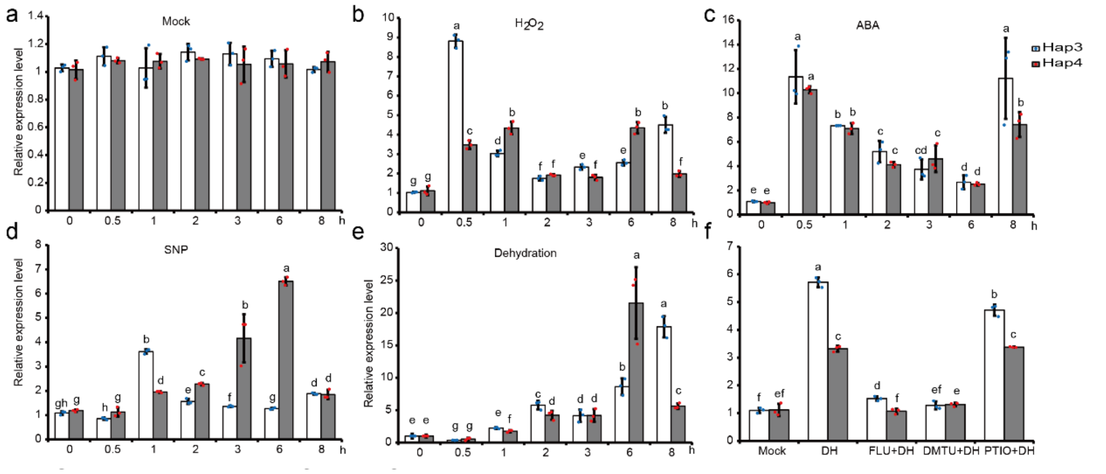

Supplementary Fig. 6. The effects of ABA, H_2O_2 and NO on the expression of *BnaA9.NF-YA7* in Hap3 and Hap4 were studied by RT–qPCR. a, Leaves treated with water as a control. **b,** 10 mM H_2O_2. **c,** 100 µM ABA. **d,** 100 µM sodium nitroprusside (SNP). **e,** Dehydration. **f,** Dependence on abscisic acid (ABA), H_2O_2 and NO. Leaves were treated with 150 µM fluridone (FLU), 5 mM dimethylthiourea (DMTU) or 200 µM 2-phenyl-4,4,5,5-tetramethylimidazoline-1-oxyl-3-oxide (PTIO) for 2 h, followed by transfer to a hood for 2 h for dehydration treatment (DH). The values are means ± SD (n = 3 biological replicates). The lowercase letters indicate significant differences ($P < 0.05$, One-way ANOVA followed by two-tailed LSD test).

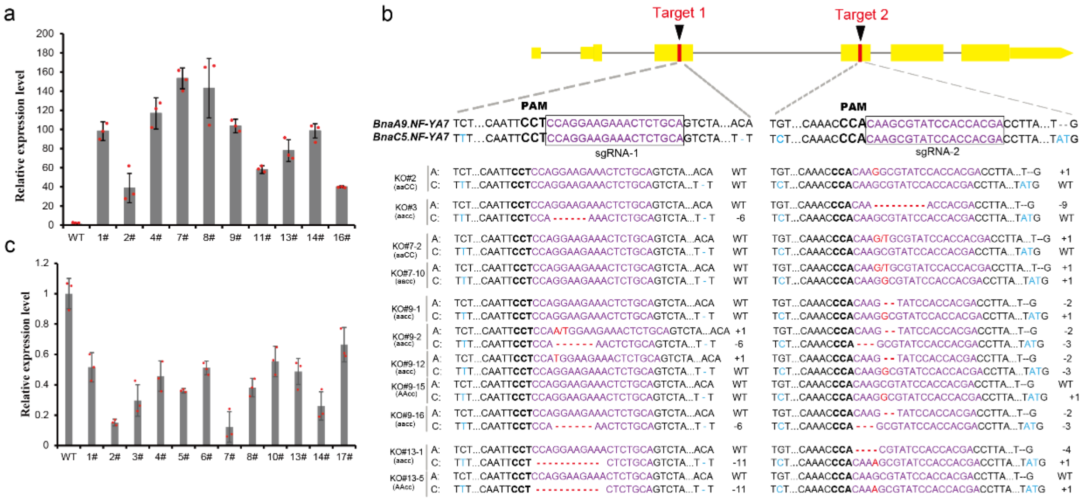

Supplementary Fig. 7. Characterization of *BnaA9.NF-YA7* overexpression, knockout, and RNA interference plants. a, Expression analysis of BnaA9.NF-YA7-OE plants. The values are means ± SD (n = 3 biological replicates). **b,** Sequences of homozygous CRISPR–Cas9 knockout lines with deletions or insertions in the target site. The gene model and the wild-type sequence are shown at the top. The target site is highlighted in green, and the protospacer-adjacent motif (PAM) sequence is indicated by black bold font. The red broken lines represent deletions. **c,** Expression analysis of BnaA9.NF-YA7-RNAi plants. The values are means ± SD (n = 3 biological replicates).

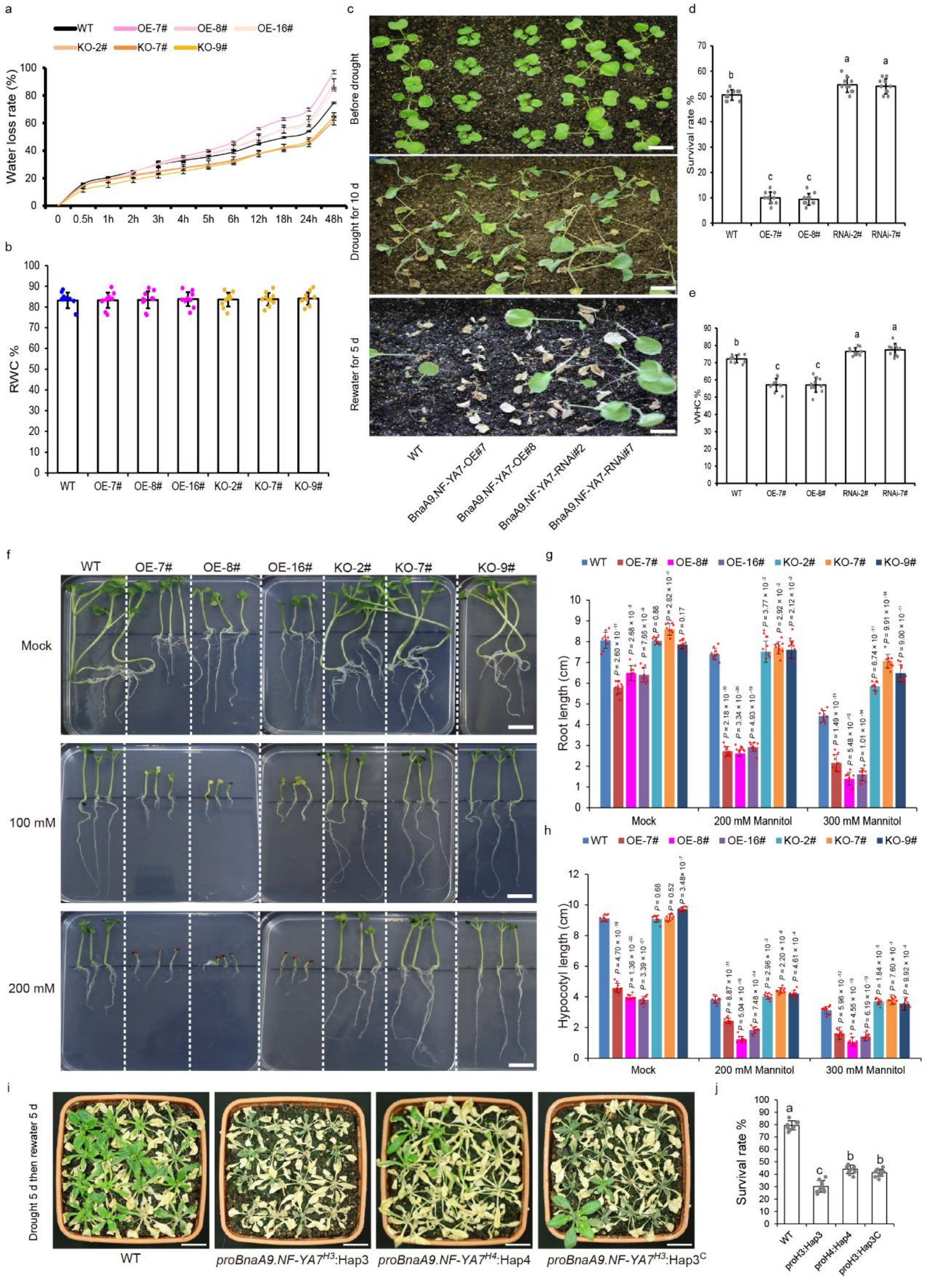

Supplementary Fig. 8. *BnaA9.NF-YA7* negatively regulates *B. napus* drought tolerance. (**a** and **b**) Comparison of the water loss rate (**a**) and relative water content (**b**) between the wild-type and *BnaA9.NF-YA7* transgenic lines. The values are means ± SD (n = 3 biologically independent experiments in **a** and n =10 biological replicates in **b**). (**c-e**) Comparison of phenotype (**c**), survival rate (**d**), and water holding capacity (**e**) among wild-type, BnaA9.NF-YA7-OE, and BnaA9.NF-YA7-RNAi transgenic lines

under well-watered, drought-stress, or rewatering conditions. The values are means ± SD (n = 10 biologically independent experiments). The lowercase letters indicate significant differences ($P < 0.05$, One-way ANOVA followed by two-tailed LSD test). **f,** Phenotypes of different genotypes grown on half-strength MS phytoagar medium containing 0, 100, or 200 mM D-mannitol for 14 days under LD conditions. White bars = 2 cm. (**g** and **h**) Quantification of root length (**g**) and hypocotyl length (h) after 14 days of D-mannitol treatment. The values are means ± SD (n = 10 biologically independent plants). P values were calculated with the two-tailed Student's t-test. (**i** and **j**) Comparison of phenotype (**i**) and survival rate (**j**) among wild-type, *pBnaA9.NF-YA7*^{H3}:Hap3, *pBnaA9.NF-YA7*^{H4}:Hap4, and *pBnaA9.NF-YA7*^{H3}:Hap3^{C} *Arabidopsis* transgenic lines after drought stress. 7-day-old *Arabidopsis* seedlings were transferred to pots, grown for 3 weeks under normal conditions, and subjected to drought conditions by withholding water from the seedlings for 5 days. The values are means ± SD (n = 10 biologically independent experiments). The lowercase letters indicate significant differences ($P < 0.05$, One-way ANOVA followed by two-tailed LSD test). Source data are provided as a Source Data file.

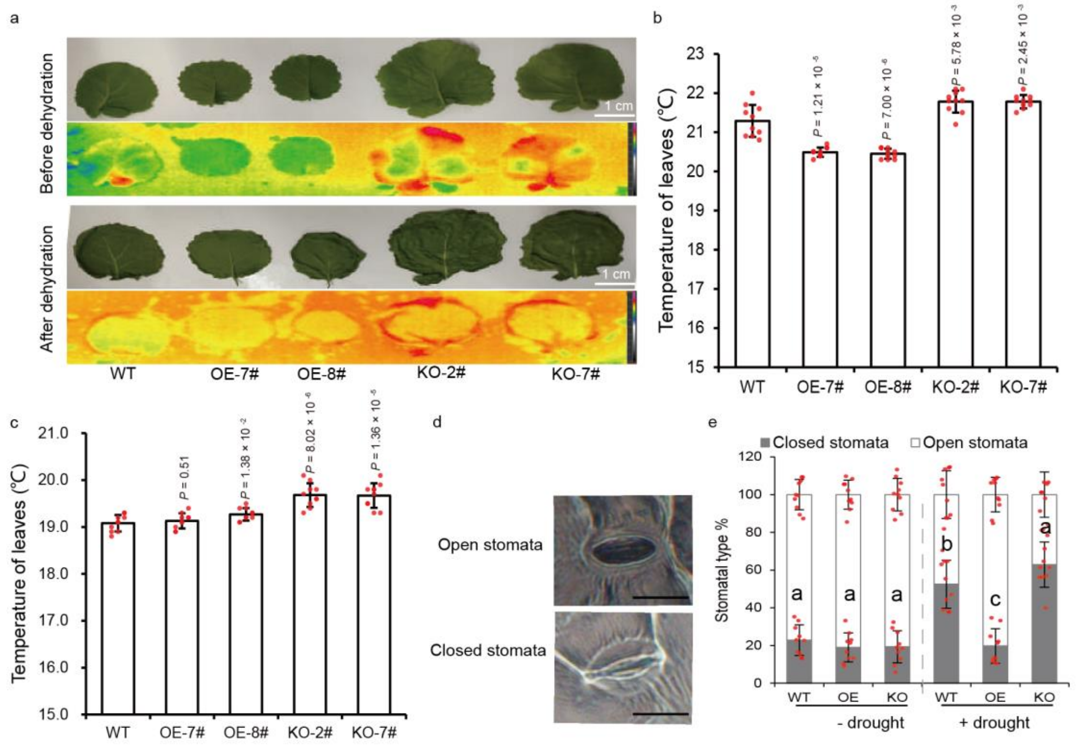

Supplementary Fig. 9. *BnaA9.NF-YA7* affects the water loss rate. a, Infrared thermal images of the WT, BnaA9.NF-YA7-OE, and BnaA9.NF-YA7-KO leaves before and after 2 h of air dehydration. The false-color scale in infrared thermal images represents temperature according to color, with red representing higher temperatures and blue representing lower temperatures. Leaves with a higher transpiration rate appear blue. Scale bars = 1 cm. (**b** and **c**) The leaf surface temperature of WT, BnaA9.NF-YA7-OE, and BnaA9.NF-YA7-KO before (**b**) and after 2 h of air dehydration (**c**) in (**a**). The values are means ± SD (n = 10 biological replicates). P values were calculated with the two-tailed Student's t-test. **d,** Microscopy images of stomatal opening in four-week-old WT plants before drought stress. Scale bars = 10 µm. **e,** Stomata type (%) in WT, BnaA9.NF-YA7-OE, and BnaA9.NF-YA7-KO leaves before and after drought stress. The values are means ± SD (n = biologically independent experiments). The lowercase letters indicate significant differences (P < 0.05, One-way ANOVA followed by two-tailed LSD test).

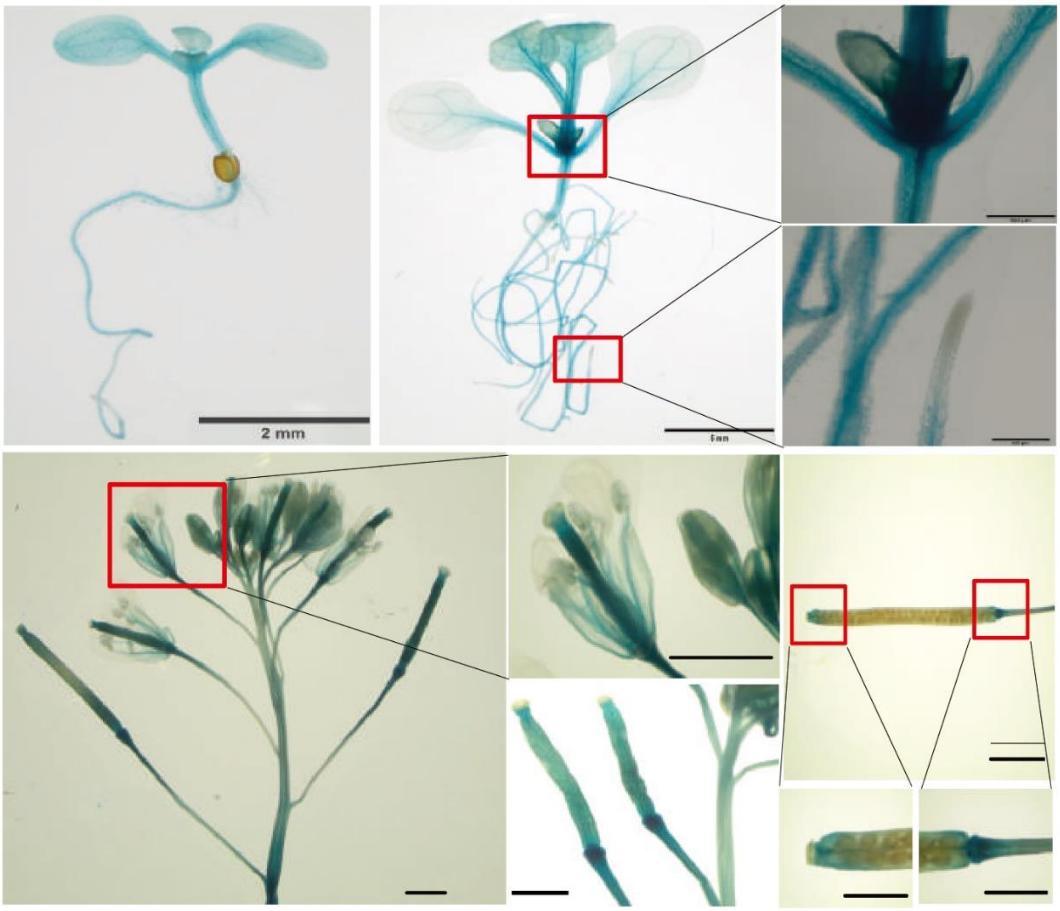

Supplementary Fig. 10. Tissue-specific expression of *BnaA9.NF-YA7*. GUS staining of *proBnaA9.NF-YA7*^{Hap3}:GUS transgenic plants at different growth stages. Bars = 2 or 5 mm.

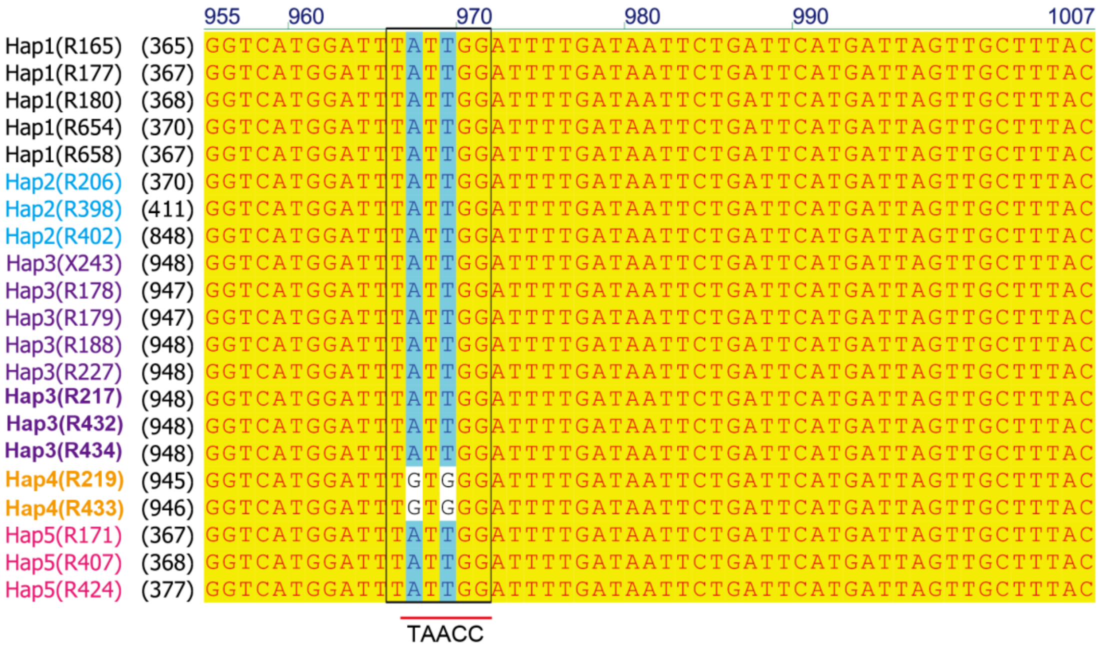

Supplementary Fig. 11. Sequence alignment results of five haplotypes around CCAAT elements. The black solid wireframe represents the CCAAT box cis-regulatory element locus.

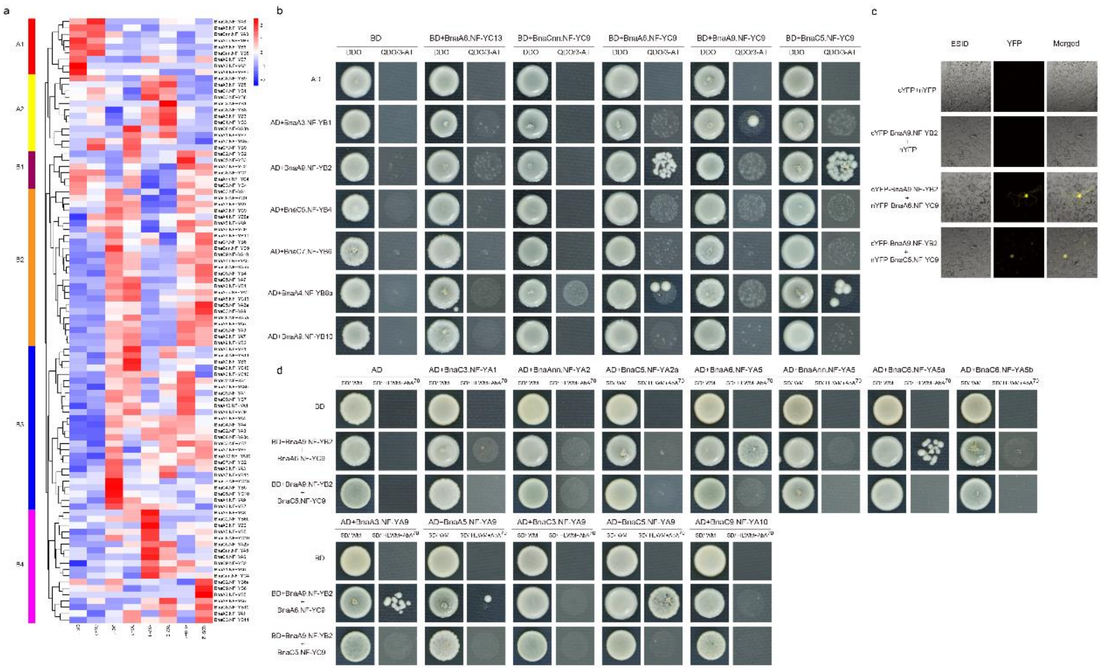

Supplementary Fig. 12. Identification of the *BnaA9.NF-YA7* upstream regulatory factor BnaNF-Y trimer. a, Heatmap of the expression profiles of BnaNF-Y family genes under drought stress at the seedling stage. **b,** The Y2H assay between six Bna.NF-YBs and five Bna.NF-YCs from B2 subgroup. **c**, The BiFC assay showed that BnaA9.NF-YB2 interacts with BnaA6.NF-YC9 and BnaC5.NF-YC9 *in vivo*. **d,** The Y3H assay between BnaA9.NF-YB2/BnaA6.NF-YC9s and 12 Bna.NF-YAs from B2 subgroup. The results showed that BnaA6.NF-YA5 and BnaC5.NF-YA9 can interact with the BnaA9.NF-YB2/BnaA6.NF-YC9 dimer to form a trimer. Source data are provided as a Source Data file.

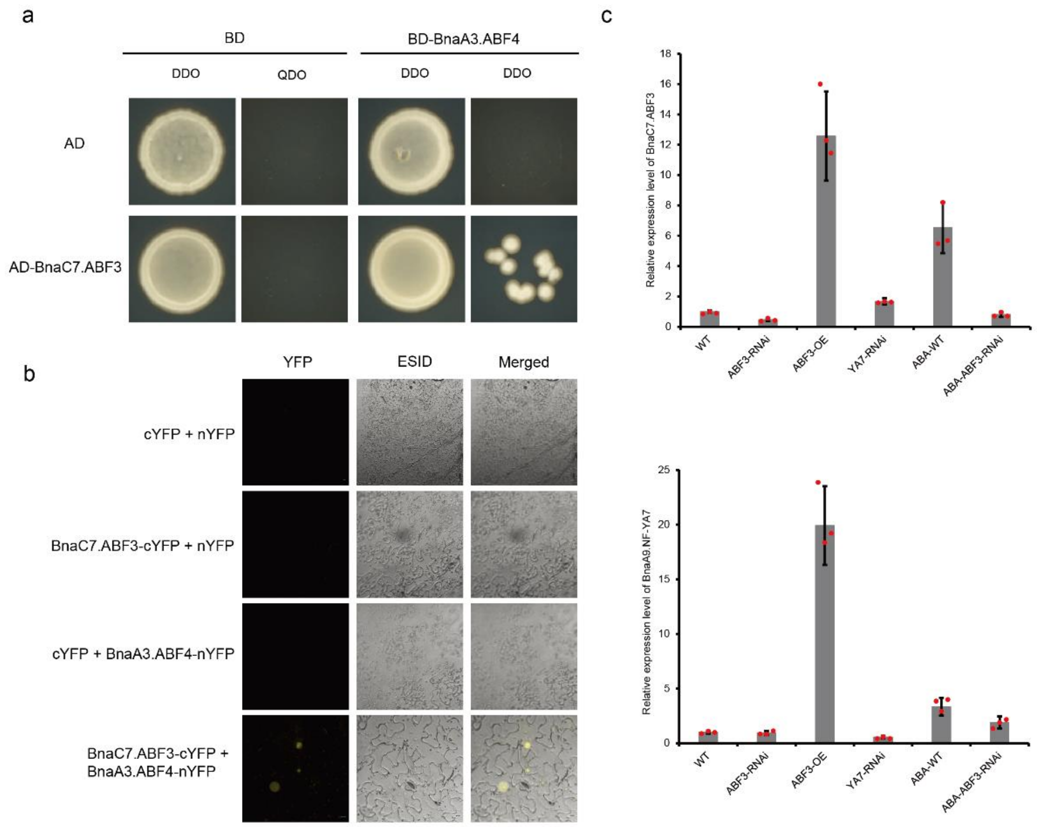

Supplementary Fig. 13. Expression patterns of *BnaA9.NF-YA7* and *BnaC7.ABF3* in their transgenic plants. a, The Y2H assay indicated that BnaC7.ABF3 and BnaA3.ABF4 interacts *in vitro*. **b,** The BiFC assay showed that BnaC7.ABF3 and BnaA3.ABF4 interacts *in vivo*. **c,** Expression levels of *BnaA9.NF-YA7* and *BnaC7.ABF3* in their transgenic plants. The values are means ± SD (n = 3 biological replicates).

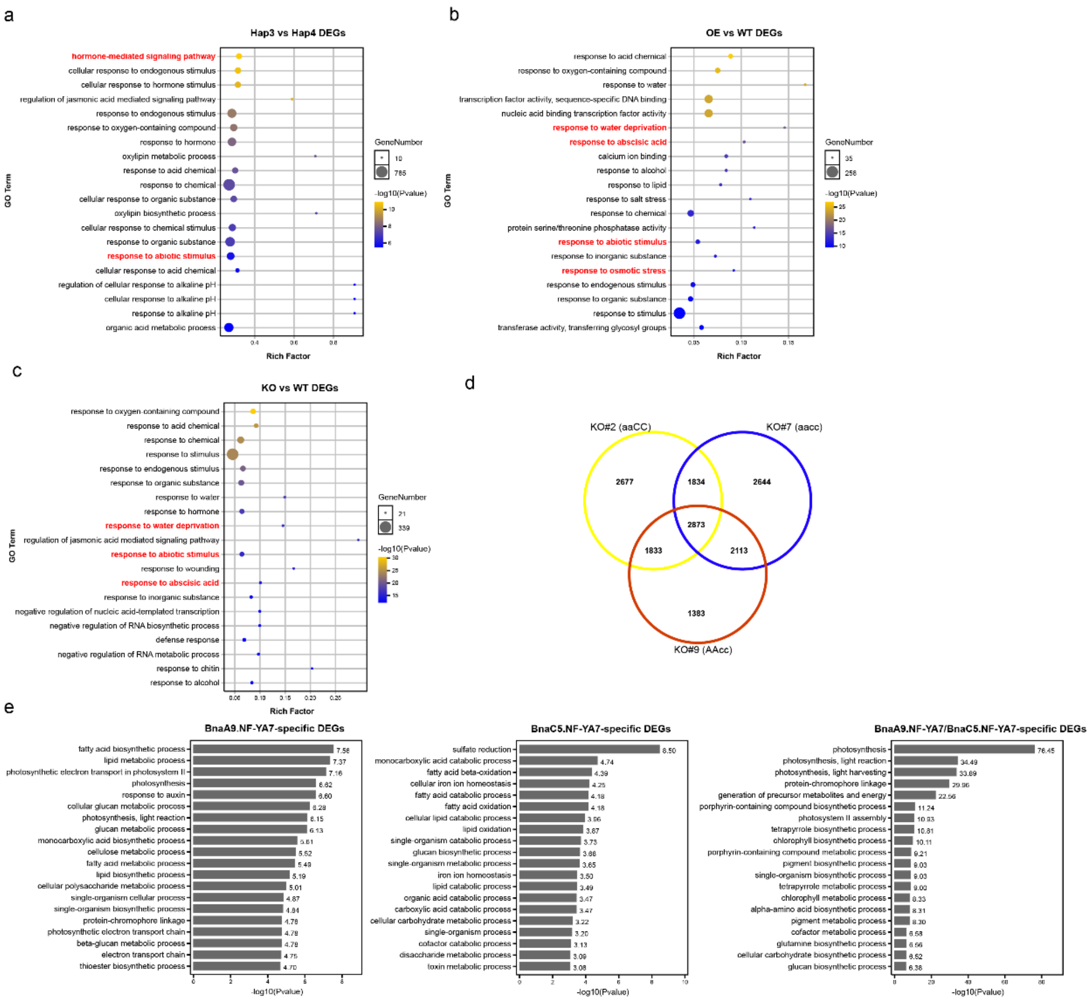

Supplementary Fig. 14. Differentially expressed genes (DEGs) in the wild-type and transgenic lines. (**a-c**) Gene Ontology (GO) analyses were performed to categorize the functions of DEGs between Hap3 and Hap4 (**a**), WT and BnaA9.NF-YA7-OE (**b**), and WT and BnaA9.NF-YA7-KO (**c**). **d,** Venn diagrams of the DEGs among aaCC, aacc, and AAcc mutants. **e,** Enriched KEGG pathways in the set of BnaA9.NF-YA7-specific DEGs BnaC5.NF-YA7-specific DEGs, and BnaA9.NF-YA7/BnaC5.NF-YA7-specific DEGs.

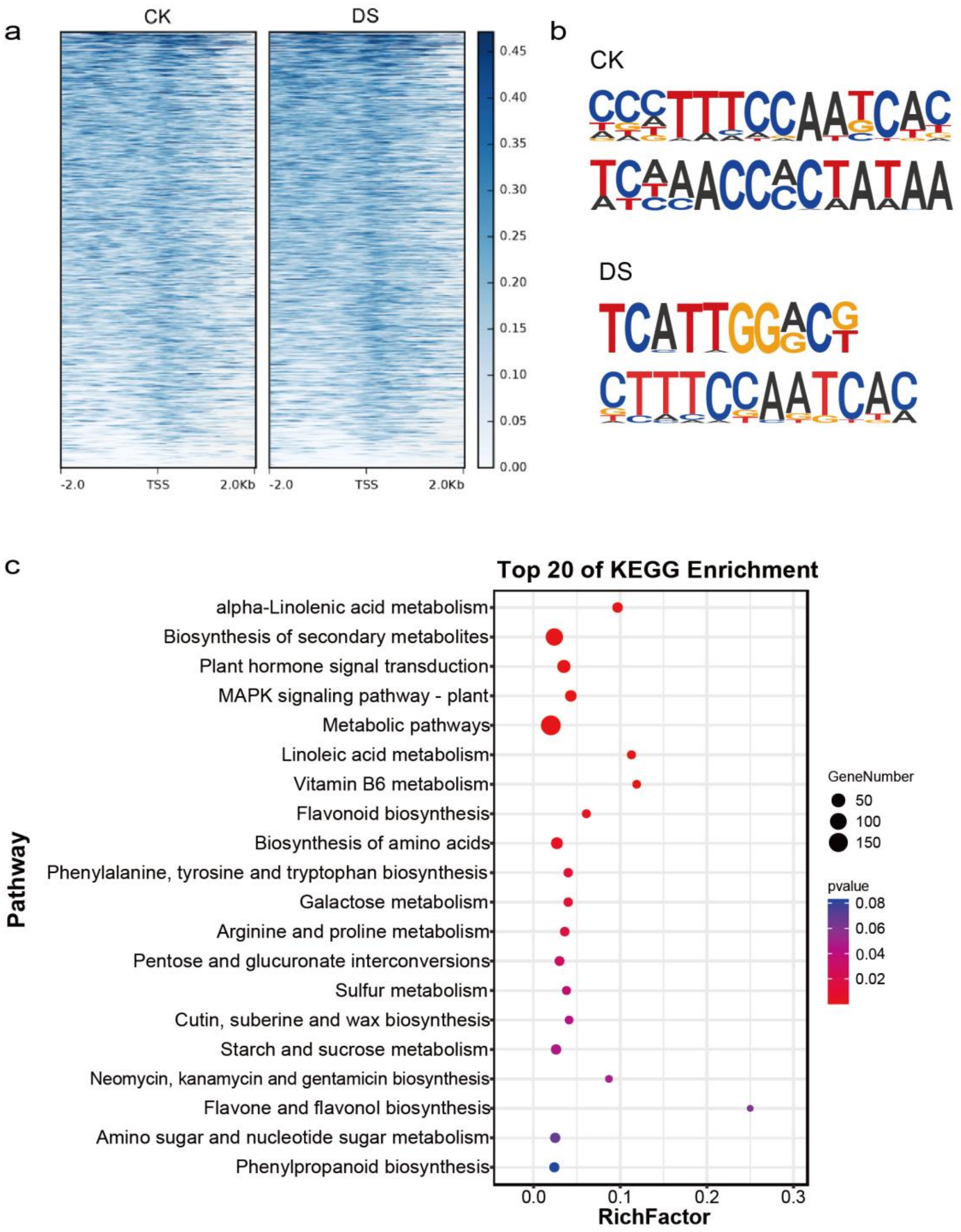

Supplementary Fig. 15. Identification of stress-related genes controlled by *BnaA9.NF-YA7*. a, Density heatmap showing enrichment of ChIP-Seq signals of BnaA9.NF-YA7 in the 2 kb around the TSS regions. **b,** Consensus sequence motifs enriched within BnaA9.NF-YA7 sites using de novo motif analysis before (CK) and after drought treatment (DS). **c,** KEGG-enriched pathways in the set of common DEGs marked in red in the Venn diagram (Fig. 5a).

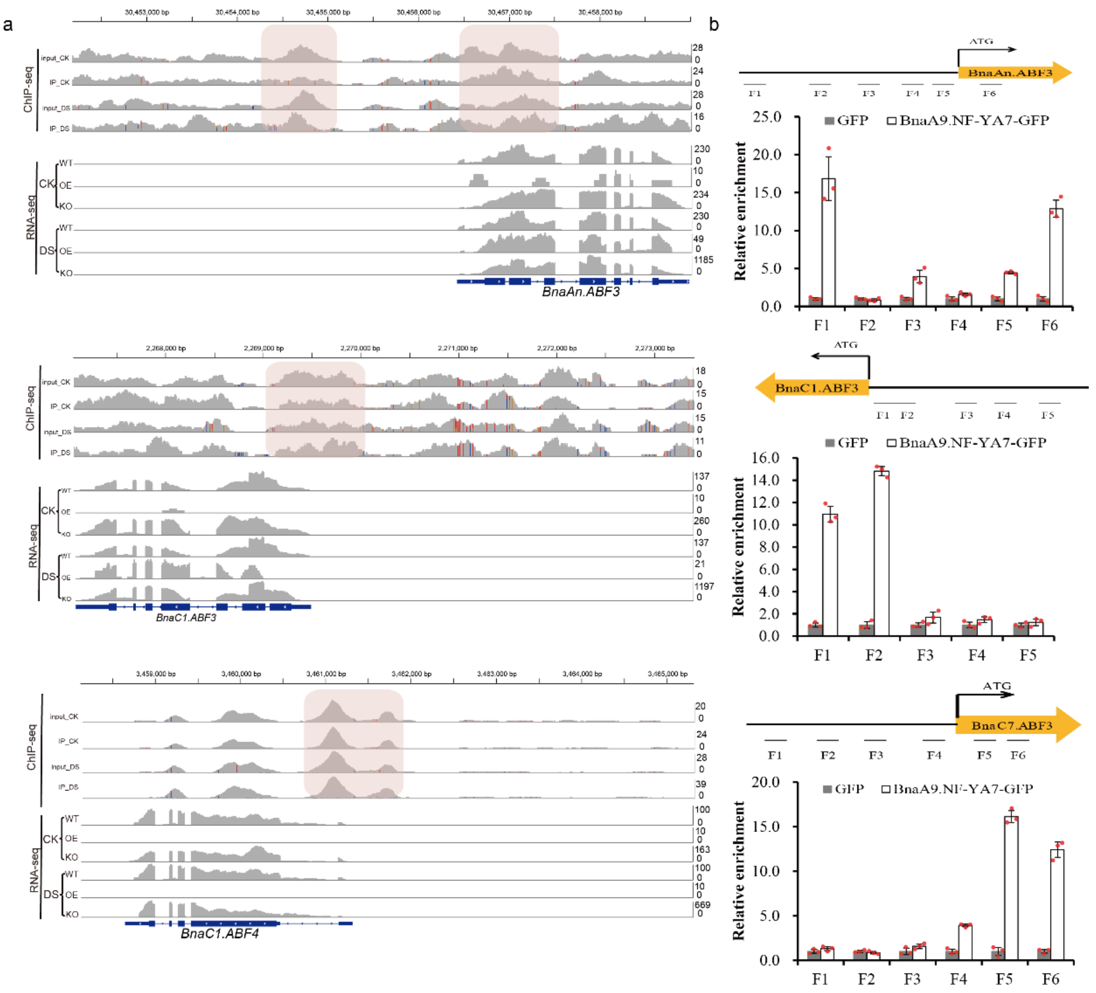

Supplementary Fig. 16. *BnaA9.NF-YA7* directly targets *BnaABF3/4*s. a, Genome browser view of normalized ChIP-seq tags from the ChIP-seq data and normalized RNA-seq tags from the RNA-seq data for *BnaA9.NF-YA7* showing *BnaAn.ABF3*, *BnaC1.ABF3*, and *BnaC1.ABF4* bind to *BnaA9.NF-YA7*. **b,** Validation of ChIP-seq data by ChIP–qPCR showing *BnaA9.NF-YA7* binding to the promoter region of *BnaABF3/4*. The values are means ± SD (n = 3 biological replicates). Source data are provided as a Source Data file.

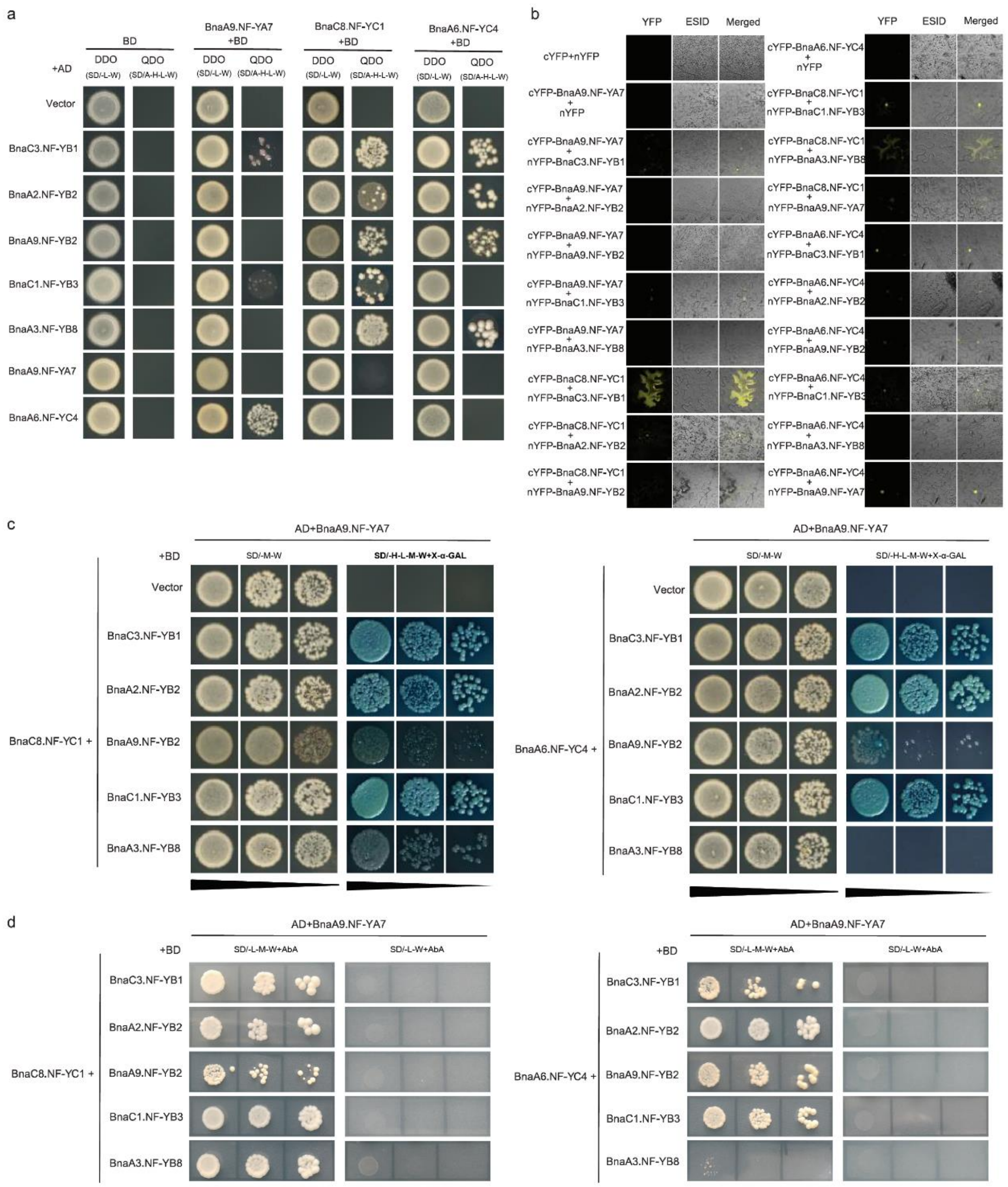

Supplementary Fig. 17. Identification of BnaNF-YB/YC dimers interacting with BnaA9.NF-YA7. a, The Y2H assay indicated that BnaNF-YBs and BnaNF-YCs interact *in vitro*. **b,** The BiFC assay showed that BnaNF-YBs and BnaNF-YCs interact *in vivo*. (**c** and **d**) The Y3H assay showed that BnaA9.NF-YA7 interacts with the BnaNF-YB/YC dimer to form a trimer (**c**), and the 3rd protein acts as a bridge for bait–prey interactions (**d**).

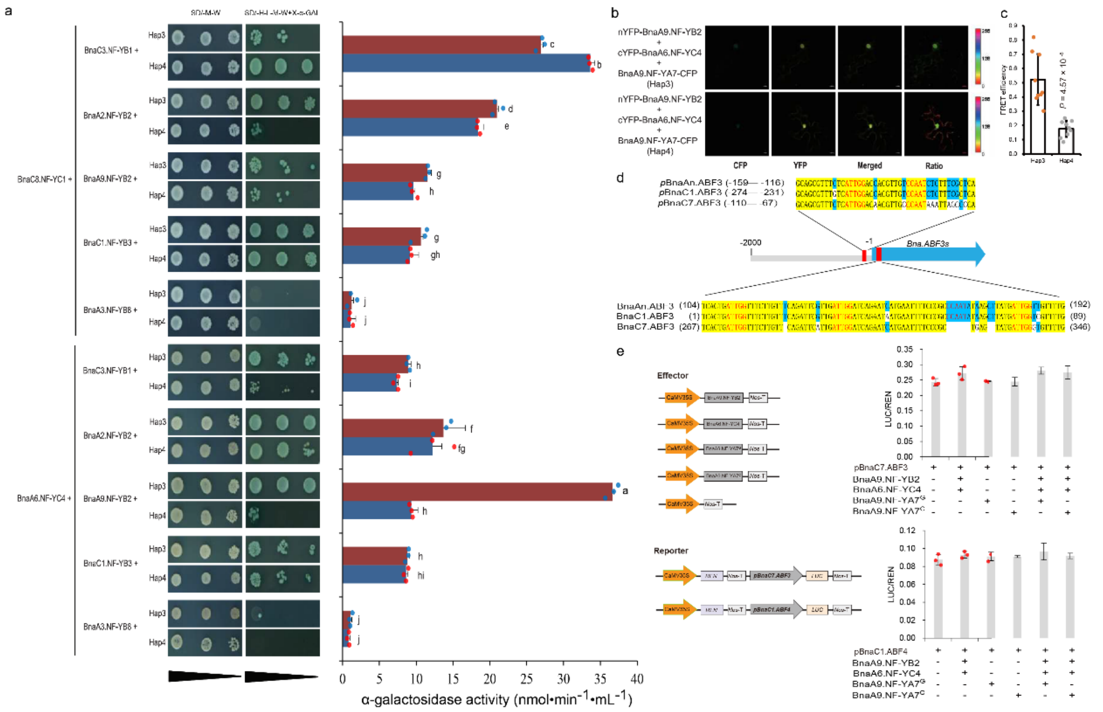

Supplementary Fig. 18. BnaA9.NF-YA7 can interact with BnaA9.NF-YB2/BnaA6.NF-YC4 dimer to form a trimer *in vitro* and *in vivo*. a, Y3H showed that BnaA9.NF-YA7 can interact with the NF-YB/NF-YC dimer to form a trimer *in vitro*. The left histogram represents the α-galactosidase activity activated by different trimer combinations. The values are means ± SD (n = 3 biological replicates). The lowercase letters indicate significant differences ($P < 0.05$, One-way ANOVA followed by two-tailed LSD test). (**b** and **c**) The fluorescence resonance energy transfer assay showed that BnaA9.NF-YA7 can interact with BnaA9.NF-YB2/BnaA6.NF-YC4 *in vivo* (**b**) and BnaA9.NF-YA7^{G} (Hap3) has a higher fluorescence ratio than BnaA9.NF-YA7^{C} (Hap4) (**c**). The values are means ± SD (n = 9 biological replicates). P values were calculated with the two-tailed Student's t-test. **d,** CCAAT element near the TSS or within the coding region of *BnaAn.ABF3*, *BnaC1.ABF3*, and *BnaC7.ABF3*. **e,** Co-expression of BnaA9.NF-YA7$^{G/C}$ with the NF-YB/C dimer activates the expression of *BnaC7.ABF3* and *BnaC1.ABF4*. The values are means ± SD (n = 3 biological replicates). Source data are provided as a Source Data file.

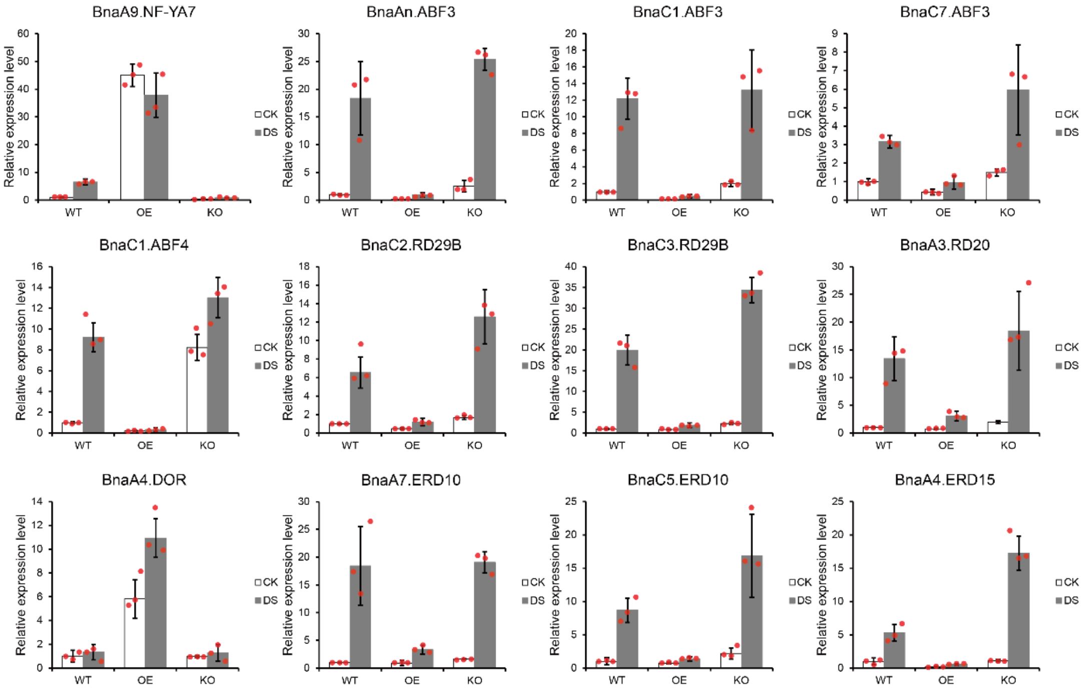

Supplementary Fig. 19. *BnaA9.NF-YA7* represses the BnaABF3/4-regulated pathway in response to drought stress. The relative transcript levels of genes were determined by RT–qPCR and normalized to the *Bna.Actin7* level. Relative expression was calculated using the $\Delta\Delta C_T$ method. Three biological replicates and three technical replicates were performed for each RT–qPCR analysis. The values are means ± SD (*n* = 3 biological replicates).

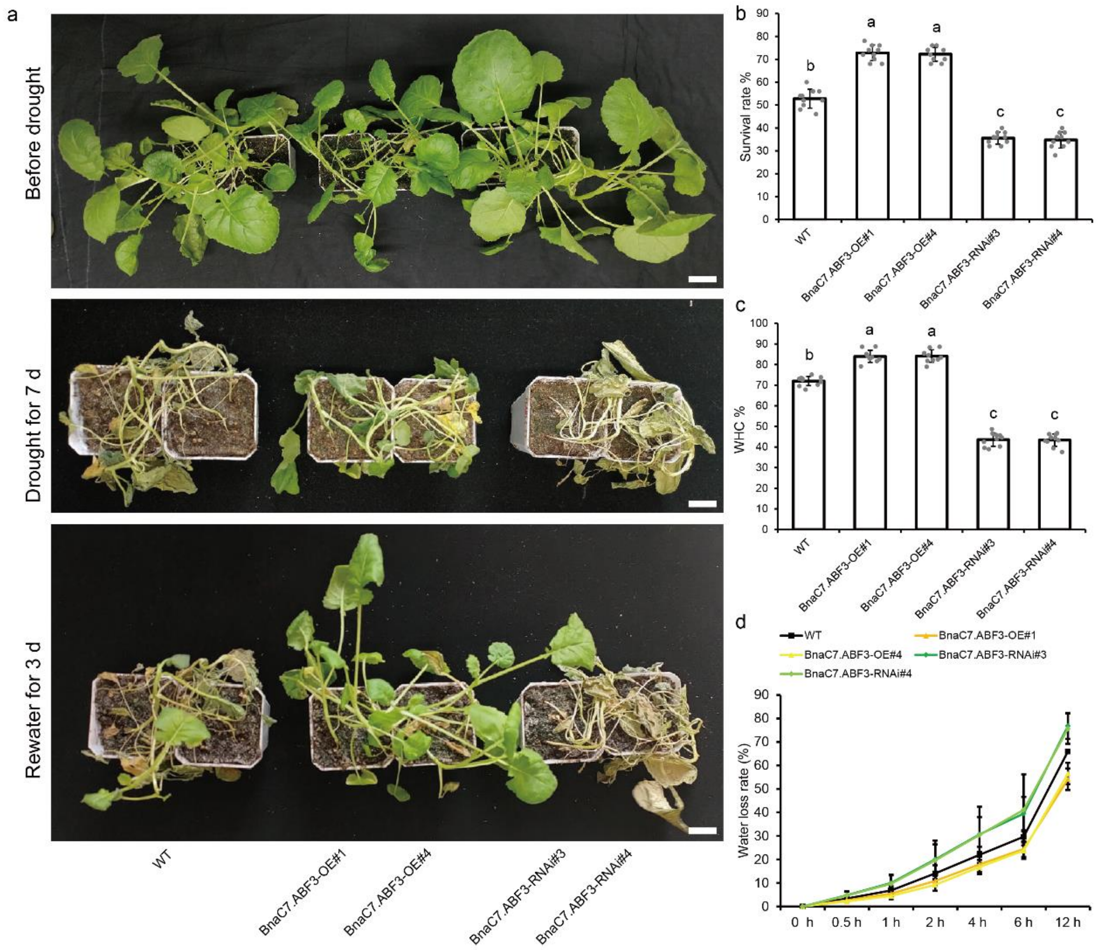

Supplementary Fig. 20. *BnaC7.ABF3* positively regulates drought tolerance in *B. napus*. a, Phenotype of *BnaC7.ABF3* transgenic plants. Four-week-old WT and *BnaC7.ABF3* transgenic plants were exposed to drought stress by withholding watering for 7 days and rewatering for 3 days. White bars = 5 cm. (**b-d**) The survival rate (**b**), water holding capacity (**c**), and water loss rate (**d**) of transgenic plants were compared with those of WT plants. The values are means ± SD (n = 10 biological replicates in **b** and **c**, and n = 3 biologically independent experiments). The lowercase letters indicate significant differences ($P < 0.05$, One-way ANOVA followed by two-tailed LSD test). Source data are provided as a Source Data file.

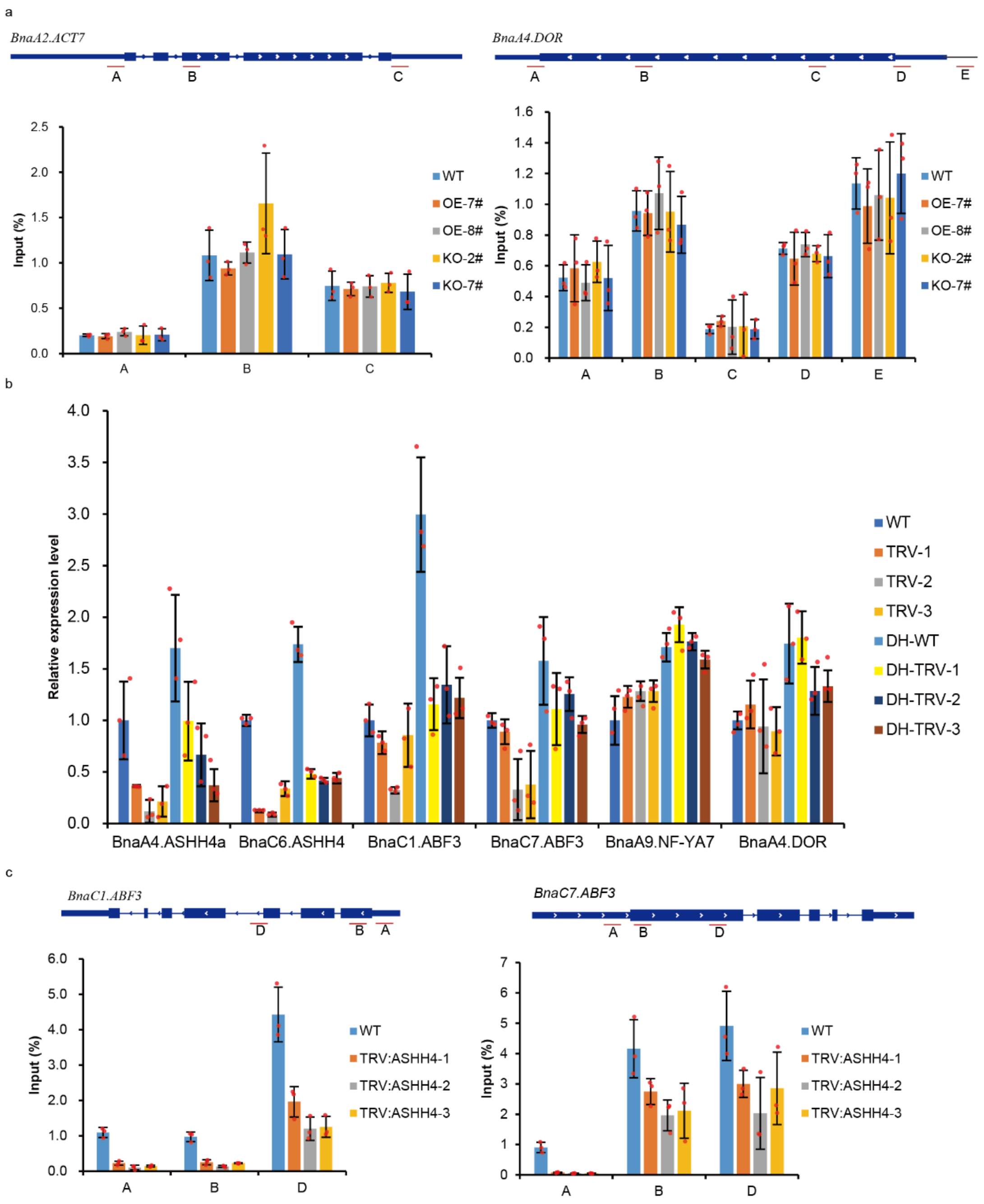

Supplementary Fig. 21. The *Bna.ASHH4* is the direct cause of the change in H3K36me3 levels change in *Bna.ABF3s*. a, *BnaA9.NF-YA7* regulates H3K36me3 levels at the *BnaA2.ACT7* locus and *BnaA4.DOR* locus under drought stress. The values are means ± SD (*n* = 9 data elements from 3 biological replicates). **b,** Determination of expression levels of related genes in TRV:Bna.ASHH4 plants by RT–qPCR. The values are means ± SD (*n* = 3 biological replicates). **c,** H3K36me3 levels in TRV:Bna.ASHH4 plants in the *BnaC1.ABF3* locus and *BnaC7.ABF3* locus. The values are means ± SD (*n* = 3 biological replicates). Source data are provided as a Source Data file.

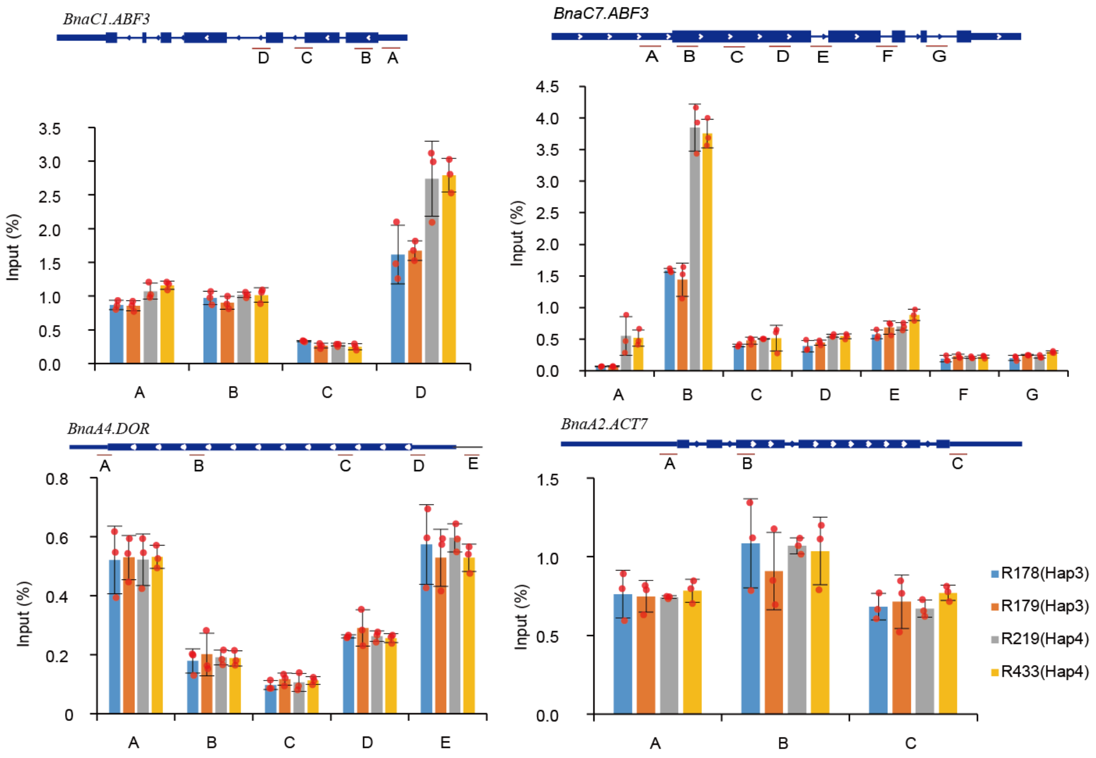

Supplementary Fig. 22. Comparison of H3K36me3 levels between Hap3 and Hap4 under drought stress. ChIP-qPCR was performed using H3K36me3 antibody. The x-axis denotes different genetic regions of *BnaC1.ABF3*, *BnaC7.ABF3*, *BnaA4.DOR*, and *BnaA2.ACT7* locus. The values are means ± SD (*n* = 3 biological replicates). Source data are provided as a Source Data file.

Supplementary Table 1. Potential regulatory genes of BnaA9.NF-YA7 screened by yeast one-hybrid.

Gene_ID	*Arabidopsis* gene name	Description
BnaC04g48630D	*PIF4*	phytochrome interacting factor 4
BnaA03g35190D	*ABF4*	ABRE binding factor 4 (ABF4)
BnaA05g08020D	*ABI5*	ABA INSENSITIVE 5 (ABI5)
BnaA09g00170D	*GBF2*	G-box binding factor 2 (GBF2)
BnaC03g23970D	*PIF4*	phytochrome interacting factor 4
BnaAnng26550D	*ABF3*	abscisic acid responsive elements-binding factor 3 (ABF3)
BnaC01g04330D	*ABF3*	abscisic acid responsive elements-binding factor 3 (ABF3)
BnaC01g19550D	*RD26*	RESPONSIVE TO DESICCATION 26 (RD26)
BnaC04g53290D	*SOC1*	AGAMOUS-like 20 (AGL20)
BnaC05g33570D	*ABF4*	ABRE binding factor 4 (ABF4)
BnaC07g44670D	*ABF3*	abscisic acid responsive elements-binding factor 3 (ABF3)
BnaC08g42660D	*phyA*	phytochrome A (PHYA)
BnaC09g05450D	*ARF2*	auxin response factor 2 (ARF2)
BnaC09g05770D	*WRKY18*	WRKY DNA-binding protein 18 (WRKY18)
BnaC09g06950D	*ATHB25*	homeobox protein 25 (HB25)
BnaC09g10430D	*AT2G20400*	myb-like HTH transcriptional regulator family protein
BnaC09g20970D	*IDD4*	indeterminate(ID)-domain 4 (IDD4)
BnaC09g34140D	*At5g58900*	Homeodomain-like transcriptional regulator
BnaC09g40660D	*RVE1*	REVEILLE 1 (RVE1)
BnaC09g46670D	*TGA4*	TGACG motif-binding factor 4 (TGA4)
BnaC09g49710D	*At5g05790*	Duplicated homeodomain-like superfamily protein
BnaC09g49920D	*DREB2A*	DRE-binding protein 2A (DREB2A)
BnaCnng01510D	*NTM2*	NAC domain containing protein 69 (NAC069)
BnaCnng01910D	*GBF2*	G-box binding factor 2 (GBF2)
BnaCnng09850D	*ANAC032*	NAC domain containing protein 32 (NAC032)
BnaCnng16520D	*At1g49010*	Duplicated homeodomain-like superfamily protein
BnaCnng19880D	*phyA*	phytochrome A (PHYA)
BnaCnng33070D	*RAV1*	related to ABI3/VP1 1 (RAV1)
BnaCnng36880D	*SOC1*	AGAMOUS-like 20 (AGL20)
BnaCnng37790D	*RAV1*	related to ABI3/VP1 1 (RAV1)
BnaCnng47140D	*AT5G56840*	myb-like transcription factor family protein
BnaCnng54100D	*ARF2*	auxin response factor 2 (ARF2)
BnaCnng66020D	*WRKY33*	WRKY DNA-binding protein 33 (WRKY33)
BnaCnng72050D	*ANAC020*	NAC domain containing protein 20 (NAC020)

Supplementary Table 2. Primers used for RT-qPCR in this work.

Gene ID	Primer (from 5' to 3')	
	**Forward**	**Revese**
BnaA9.NF-YA7	ATGGGAGTCCAGCAACAATG	CGTGATGCTCCTCTTCCTTC
BnaC5.NF-YA7	CGAAGGCAATCATGTTGAAGAA	ACCGCTACGTGTATATGTCATT
BnaA3.NF-YB8	CAAGACAGGTTTCTTCCGATTG	GTCCTTAGCGATCTTCCCATTA
AtNF-YA7	TTCCTTTACCATCTGATGCAGT	GAGATTCATGCAAATACGGCTT
AtABF3	GATGTGGTTAACCGTTCTCAAC	CAGCTTGCAGTAGATTGTTGTT
AtABF4	TTCCTAAACAAGCAAACGTAGC	CGATTAAGCACATACGGAACTG
BnaC1.ABF3	GCCGATTTGACTAGATCAACAC	CGTCCTAGAAAGCAACATCAAG
BnaAn.ABF3	ACTTGAAGGATCCAAAGCTTGA	CAGCTGTCCAAATGTTCTTCAA
BnaC7.ABF3	AACATGTGGAGAGGCTTGATAA	AGCTTGAATTTTCAGCAGATCC
BnaC2.RD29B	TGGAAAATGGATTTGACACGTC	TTGCCATCAATGCCATGATTAG
BnaC3.RD29B	TGAACCTGAGCCAATACAAGAT	GCATCTGATGATACTCTCTGCT
BnaA9.RAB18	TTAAACTCTGGGACAAACG	AGCGATGCCTCTTACACTC
BnaA3.RD20	TTGGCTAGAGCATTAGTAGCTC	ACGCTAGGTTTACGATTAAGCT
BnaA7.ERD10	AGGAGATGAAAAGAAGGGAGTG	ACGACTTGAGAATCATCTGGTT
BnaC5.ERD10	AACTGTTCAGGCGAAAACTATG	TCAAACAGTCCACGATCCTTAA
BnaA4.ERD15	TGCCAAGTATAACCAGAAGAGG	TTTTATTGCTTTCTCCAGACGC
BnaA4.DOR	TCTTCTGTTATGGAGATGACCG	CGACTTATTCATCGACAACACC
BnaA4.ASHH4a	GGCTAAGTCTACTAAAGCCCAT	AATGAGCGGCAGTTTTATTACC
BnACT7	TGGGTTTGCTGGTGACGAT	TGCCTAGGACGACCAACAATACT
AtActin	CCACATGCTATTCTGCGTTTGGACC	CATCCCTTACGATTTCACGCTCTGC
