# Supplementary material for: Natural variation in BnaA9.NF-YA7 contributes to drought tolerance in Brassica napus L
Source: Nat Commun. 2024 Mar 7;15:2082. doi: 10.1038/s41467-024-46271-2 (PMC10920887; doi:10.1038/s41467-024-46271-2)
Supplement: Supplementary file 3 — Description of Additional Supplementary Files [file 41467_2024_46271_MOESM3_ESM.pdf]

Description of Additional Supplementary Files:

Supplementary Data 1: Natural variation information within the *BnaA9.NF-YA7* gene and promoter regions.

Supplementary Data 2: GO term enrichment analysis of differentially expressed genes between Hap3 and Hap4.

Supplementary Data 3: GO term enrichment analysis of differentially expressed genes between OE and WT.

Supplementary Data 4: GO term enrichment analysis of differentially expressed genes between KO and WT.

Supplementary Data 5: The binding peaks and neighboring genes were identified by ChIP-Seq assays.

Supplementary Data 6: Expression data of genes related to the plant hormone signal transduction pathway.

Supplementary Data 7: Primers used for plasmid construction in this work.

Supplementary Data 8: Primers used for ChIP–qPCR in this work.
