# Supplementary material for: Natural variation in BnaA9.NF-YA7 contributes to drought tolerance in Brassica napus L
Source: Nat Commun. 2024 Mar 7;15:2082. doi: 10.1038/s41467-024-46271-2 (PMC10920887; doi:10.1038/s41467-024-46271-2)
Supplement: Supplementary file 12 — Reporting Summary [file 41467_2024_46271_MOESM12_ESM.pdf]

nature portfolio

Corresponding author(s): Liezhao Liu

Last updated by author(s): Feb 14, 2024

Reporting Summary

Nature Portfolio wishes to improve the reproducibility of the work that we publish. This form provides structure for consistency and transparency in reporting. For further information on Nature Portfolio policies, see our Editorial Policies and the Editorial Policy Checklist.

Statistics

For all statistical analyses, confirm that the following items are present in the figure legend, table legend, main text, or Methods section.

n/a	Confirmed	
☐	☒	The exact sample size (*n*) for each experimental group/condition, given as a discrete number and unit of measurement
☐	☒	A statement on whether measurements were taken from distinct samples or whether the same sample was measured repeatedly
☐	☒	The statistical test(s) used AND whether they are one- or two-sided<br>*Only common tests should be described solely by name; describe more complex techniques in the Methods section.*
☒	☐	A description of all covariates tested
☐	☒	A description of any assumptions or corrections, such as tests of normality and adjustment for multiple comparisons
☐	☒	A full description of the statistical parameters including central tendency (e.g. means) or other basic estimates (e.g. regression coefficient) AND variation (e.g. standard deviation) or associated estimates of uncertainty (e.g. confidence intervals)
☐	☒	For null hypothesis testing, the test statistic (e.g. *F*, *t*, *r*) with confidence intervals, effect sizes, degrees of freedom and *P* value noted<br>*Give P values as exact values whenever suitable.*
☒	☐	For Bayesian analysis, information on the choice of priors and Markov chain Monte Carlo settings
☒	☐	For hierarchical and complex designs, identification of the appropriate level for tests and full reporting of outcomes
☒	☐	Estimates of effect sizes (e.g. Cohen's *d*, Pearson's *r*), indicating how they were calculated

Our web collection on statistics for biologists contains articles on many of the points above.

Software and code

Policy information about availability of computer code

Data collection	RNA-seq and ChIP-seq data were generated from illumina Hiseq 4000 and high coverage data from Hiseq X ten sequencing platform.
Data analysis	For RNA-seq analysis, we used TopHat (version 2.1.1), IGV (version 2.10.0), DESeq2 (version 1.6.3).<br>For Association analysis: GAPIT (version), CMplot (version)<br>For Phylogenetic analysis: ClustalX (version 2.1.0), MEGA (version 5.0).<br>Others: ImageJ (version 1.8.0)

For manuscripts utilizing custom algorithms or software that are central to the research but not yet described in published literature, software must be made available to editors and reviewers. We strongly encourage code deposition in a community repository (e.g. GitHub). See the Nature Portfolio guidelines for submitting code & software for further information.

Data

Policy information about availability of data

All manuscripts must include a data availability statement. This statement should provide the following information, where applicable:

- Accession codes, unique identifiers, or web links for publicly available datasets
- A description of any restrictions on data availability
- For clinical datasets or third party data, please ensure that the statement adheres to our policy

Sequencing data associated with this study have been deposited in the NCBI Sequence Read Archive under BioProject accession numbers PRJNA977421,

PRJNA978264, and PRJNA978979. The primers used in this work are listed in Supplementary Table 2, Supplementary Data 7, and Supplementary Data 8. Other data needed to evaluate the conclusions in the paper are presented in the paper or the supplementary materials. Source data are provided with this paper.

Research involving human participants, their data, or biological material

Policy information about studies with human participants or human data. See also policy information about sex, gender (identity/presentation), and sexual orientation and race, ethnicity and racism.

Reporting on sex and gender	Not involved.
Reporting on race, ethnicity, or other socially relevant groupings	Not involved.
Population characteristics	Not involved.
Recruitment	Not involved.
Ethics oversight	Not involved.

Note that full information on the approval of the study protocol must also be provided in the manuscript.

Field-specific reporting

Please select the one below that is the best fit for your research. If you are not sure, read the appropriate sections before making your selection.

☒ Life sciences ☐ Behavioural & social sciences ☐ Ecological, evolutionary & environmental sciences

For a reference copy of the document with all sections, see nature.com/documents/nr-reporting-summary-flat.pdf

Life sciences study design

All studies must disclose on these points even when the disclosure is negative.

Sample size	Sample size of allexperimental was determined based on the feasibility of sampling and the need of statical analysis. ALL replicates represent biologically independent samples. We used 608 rapeseed accessions to perform GWAS and phylogenetic analysis.
Data exclusions	No data was excluded from the analyses.
Replication	Conclusions drawn in this study are based on at least three independent experiments. The number of biological replicates in each experiment is indicated in the figure legends. For each materials, the border rows were removed and the middle plants were selected to conduct phenotyping.
Randomization	We always randomly selected the samples based on the genotype for experiments.
Blinding	The investigators were blinded to group allocation during data analysis.

Behavioural & social sciences study design

All studies must disclose on these points even when the disclosure is negative.

Study description	
Research sample	
Sampling strategy	
Data collection	
Timing	
Data exclusions	
Non-participation	
Randomization	

Ecological, evolutionary & environmental sciences study design

All studies must disclose on these points even when the disclosure is negative.

Study description	
Research sample	
Sampling strategy	
Data collection	
Timing and spatial scale	
Data exclusions	
Reproducibility	
Randomization	
Blinding	

Did the study involve field work? ☐ Yes ☐ No

Field work, collection and transport

Field conditions	
Location	
Access & import/export	
Disturbance	

Reporting for specific materials, systems and methods

We require information from authors about some types of materials, experimental systems and methods used in many studies. Here, indicate whether each material, system or method listed is relevant to your study. If you are not sure if a list item applies to your research, read the appropriate section before selecting a response.

Materials & experimental systems

n/a	Involved in the study	
☐	☒	Antibodies
☒	☐	Eukaryotic cell lines
☒	☐	Palaeontology and archaeology
☒	☐	Animals and other organisms
☒	☐	Clinical data
☒	☐	Dual use research of concern
☐	☒	Plants

Methods

n/a	Involved in the study	
☐	☒	ChIP-seq
☒	☐	Flow cytometry
☒	☐	MRI-based neuroimaging

Antibodies

Antibodies used	GFP (Abcam, ab290); Flag (CST, 14793S); H3K36me3 (Abcam, ab9050); anti-Histone H3 (Abcam, ab1791); H3K4me3(Abcam, ab8580); H3K27me3 (Abcam, ab6002); anti-FlAG (Beyotime Biotechnology, AF2852); anti-Myc (Beyotime Biotechnology, AF2864); anti-His (Beyotime Biotechnology, AF2876); and GST (Beyotime Biotechnology, AF0174).
Validation	Information of anti-GFP (Abcam, ab290) validation can be found at website: https://www.abcam.cn/products/primary-antibodies/gfp-antibody-ab290.html.<br>Information of anti-H3K36me3 (Abcam, ab9050) validation can be found at website: https://www.abcam.cn/products/primary-antibodies/histone-h3-tri-methyl-k36-antibody-chip-grade-ab9050.html.

Information of anti-Histone H3 (Abcam, ab1791) validation can be found at website: https://www.abcam.cn/products/primary-antibodies/histone-h3-antibody-nuclear-marker-and-chip-grade-ab1791.html.
Information of anti-H3K4me3 (Abcam, ab8580) validation can be found at website: https://www.abcam.cn/products/primary-antibodies/histone-h3-tri-methyl-k4-antibody-chip-grade-ab8580.html.
Information of anti-H3K27me3 (Abcam, ab6002) validation can be found at website: https://www.abcam.cn/products/primary-antibodies/histone-he-tri-methyl-k27-antibody-mabcam-6002-chip-grade-ab6002.html.
Information of anti-Flag (CST, 14793S) validation can be found at website: https://www.cellsignal.cn/products/primary-antibodies/dykddddk-tag-d6w5b-rabbit-mab-binds-to-same-epitope-as-sigma-s-anti-flag-m2-antibody/14793.
Information of anti-FLAG validation can be found at website: https://www.beyotime.com/product/AF2852-1ml.htm.
Information of anti-Myc validation can be found at website: https://www.beyotime.com/product/AF2864-1ml.htm.
Information of anti-His validation can be found at website: https://www.beyotime.com/product/2876-1ml.htm.
Information of anti-GST antibodies validation can be found at website: https://www.beyotime.com/product/AF0174.htm.

Eukaryotic cell lines

Policy information about cell lines and Sex and Gender in Research

Cell line source(s)

Authentication

Mycoplasma contamination

Commonly misidentified lines
(See ICLAC register)

Palaeontology and Archaeology

Specimen provenance

Specimen deposition

Dating methods

☐ Tick this box to confirm that the raw and calibrated dates are available in the paper or in Supplementary Information.

Ethics oversight

Note that full information on the approval of the study protocol must also be provided in the manuscript.

Animals and other research organisms

Policy information about studies involving animals; ARRIVE guidelines recommended for reporting animal research, and Sex and Gender in Research

Laboratory animals

Wild animals

Reporting on sex

Field-collected samples

Ethics oversight

Note that full information on the approval of the study protocol must also be provided in the manuscript.

Clinical data

Policy information about clinical studies
All manuscripts should comply with the ICMJE guidelines for publication of clinical research and a completed CONSORT checklist must be included with all submissions.

Clinical trial registration

Study protocol

Data collection

Outcomes

Dual use research of concern

Policy information about dual use research of concern

Hazards

Could the accidental, deliberate or reckless misuse of agents or technologies generated in the work, or the application of information presented in the manuscript, pose a threat to:

No	Yes	
☐	☐	Public health
☐	☐	National security
☐	☐	Crops and/or livestock
☐	☐	Ecosystems
☐	☐	Any other significant area

Experiments of concern

Does the work involve any of these experiments of concern:

No	Yes	
☐	☐	Demonstrate how to render a vaccine ineffective
☐	☐	Confer resistance to therapeutically useful antibiotics or antiviral agents
☐	☐	Enhance the virulence of a pathogen or render a nonpathogen virulent
☐	☐	Increase transmissibility of a pathogen
☐	☐	Alter the host range of a pathogen
☐	☐	Enable evasion of diagnostic/detection modalities
☐	☐	Enable the weaponization of a biological agent or toxin
☐	☐	Any other potentially harmful combination of experiments and agents

Plants

Seed stocks

The diversity panel used in this study was from a previous report (Lu et al., 2019). The B. napus transgenic lines used in this study were in the ZS 11genetic background.The GUS transgenic line harboring a 1697 bp promoter fragment was transformed into Arabidopsis (Col-0) by floral dipping.

Novel plant genotypes

The overexpression and RNAi recombinant plasmids were introduced into the B. napus cultivar ZS 11 through Agrobacterium-mediated transformation. The transgenic plants were verified by PCR, and the expression level in transgenic plants were confirmed by RT-qPCR. At least five independent lines were obtained from each transgenic line, and homozygous T3 generation plants were used for conducting the experiment.
For knockout, two 18-bp targeting sequence were synthesized and cloned into vector to produce the CRISPR-Cas9 plasmid. The CRISPR-Cas9 plasmid was introduced into the b. napus cultivar ZS 11through Agrobacterium-mediated transformation as described above. The target was amplified by PCR using primer pairs flanking the designed target site, and the PCRproduct was sequenced.

Authentication

For overexpression and RNAi lines, the expression level were confirmed by RT-qPCR. Use no less than three lines with different expression levels to observe phenotypes and exclude potential secondary effects.
For knockout, through whole genome blast, select gRNA that uniquely identify the target gene to reduce potential off target effects. Identify the target editing status through PCR product sequencing, compare the phenotypes of different editing results of individual plants, and select individuals with consistent phenotypes for the next experiment.

ChIP-seq

Data deposition

☒ Confirm that both raw and final processed data have been deposited in a public database such as GEO.

☒ Confirm that you have deposited or provided access to graph files (e.g. BED files) for the called peaks.

Data access links
May remain private before publication.

Data deposited at https://www.ncbi.nlm.nih.gov/bioproject/PRJNA978979

Files in database submission	A list data as below: Input_CK_1.fq.gz; Input_CK_2.fq.gz; IP_CK_1.fq.gz; IP_CK_2.fq.gz; Input_DS_1.fq.gz; Input_DS_2.fq.gz; IP_DS_.fq.gz; IP_DS_2.fq.gz.
Genome browser session (e.g. UCSC)	Not involved

Methodology

Replicates	Not involved
Sequencing depth	More than x20 depth for each sample
Antibodies	GFP (Abcam, ab290)
Peak calling parameters	The peak calling used the default parameters
Data quality	The peaks called in CK_GFP (24578 peaks) and DS_GFP (4210 peaks) at FDR 5%.
Software	fastp (version 0.19.11); fastqc (version v0.11.5); BWA (version 0.7.12-r1039); deepTools (version 1.5.9); MACS2 (version 2.1.0); homer findMotifsGenome.pl (version v4.9.1); Goseq, topGO (version 4.10.2); and KOBAS (version 3.0).

Flow Cytometry

Plots

Confirm that:

- [ ] The axis labels state the marker and fluorochrome used (e.g. CD4-FITC).
- [ ] The axis scales are clearly visible. Include numbers along axes only for bottom left plot of group (a 'group' is an analysis of identical markers).
- [ ] All plots are contour plots with outliers or pseudocolor plots.
- [ ] A numerical value for number of cells or percentage (with statistics) is provided.

Methodology

Sample preparation	
Instrument	
Software	
Cell population abundance	
Gating strategy	

- [ ] Tick this box to confirm that a figure exemplifying the gating strategy is provided in the Supplementary Information.

Magnetic resonance imaging

Experimental design

Design type	
Design specifications	
Behavioral performance measures	

Acquisition

Imaging type(s)	
Field strength	
Sequence & imaging parameters	
Area of acquisition	

Diffusion MRI ☐ Used ☐ Not used

Preprocessing

Preprocessing software

Normalization

Normalization template

Noise and artifact removal

Volume censoring

Statistical modeling & inference

Model type and settings

Effect(s) tested

Specify type of analysis: ☐ Whole brain ☐ ROI-based ☐ Both

Statistic type for inference

(See Eklund et al. 2016)

Correction

Models & analysis

n/a	Involved in the study
☐	☐ Functional and/or effective connectivity
☐	☐ Graph analysis
☐	☐ Multivariate modeling or predictive analysis

Functional and/or effective connectivity

Graph analysis

Multivariate modeling and predictive analysis
